# Peri-injury symptomatology as predictors of brain computed tomography (CT) scan abnormalities in mild traumatic brain injury (mTBI)

**DOI:** 10.1186/s12245-024-00754-7

**Published:** 2024-11-05

**Authors:** Sihi Vasista, Josue Saint-Fleur, Neera Kapoor, Latha Ganti

**Affiliations:** 1 Seminole High School, Sanford, FL USA; 2 Orlando College of Osteopathic Medicine, Winter, FL 34787 USA; 3 New York University’s Grossman School of Medicine, New York, NY 10016 USA; 4 Warren Alpert Medical School of Brown University, 222 Richmond Street, Providence, RI 02903 USA

**Keywords:** Mild TBI, Brain CT, Clinical decision rules

## Abstract

**Objective:**

This study aimed to identify predictors of brain CT abnormalities in patients who sustained a mild traumatic brain injury (mTBI).

**Methods:**

Retrospective observational cohort of adult patients with mTBI (Glasgow Coma Score 13–15) that occurred within the preceding 24 h.

**Results:**

2548 (91%) of the cohort had a brain CT and 698 (27%) demonstrated abnormal findings. The most frequently observed CT abnormalities were bleeding (638, 25%) and fractures (190, 7.4%). Multivariate logistic regression analysis revealed several significant predictors associated with the presence of brain CT abnormalities including older age [*P* < 0.0001], male sex [*P* < 0.0001], loss of consciousness [*P* = 0.0041], associated vomiting [*P* = 0.0011], alteration of consciousness (AOC) [*P* = 0102], and GCS score [*P* < 0.0001]. This was a robust model with an R² of 14.2%.

**Conclusion:**

In this retrospective analysis, older age, male sex, the presence of loss of consciousness or alteration in consciousness, lower GCS score, and associated vomiting were found to be significant predictors of having an abnormal brain CT. These findings highlight the importance of considering these factors when determining the necessity of brain CT scans in patients with mTBI and suggest that existing clinical decision rules may be limited. These findings may also help to inform clinical decision rules. Early identification of individuals at a higher risk of CT abnormalities may assist in appropriate management and allocation of healthcare resources.

## Introduction

In the United States, the Center for Disease Control and Prevention (CDC) documented over 200,000 hospitalizations related to traumatic brain injury (TBI) in 2020, with over 80% of these cases being classified as mild. This translates to a daily average of 586 hospitalizations and 10 deaths due to TBI, highlighting the severity of this injury [1]. TBIs contribute to almost 1/3 of all injury-related deaths [2]. Mild Traumatic Brain Injury (mTBI) is a significant health concern, characterized by neurological dysfunction following a trauma. It is usually defined by a Glasgow Coma Score of 13–15, although there is a wide variation within this range, with significant injuries noted in patient even with a GCS of 15 [3].

Sometimes referred to as post-concussive syndrome, the sequelae of mTBI includes a range of physical, cognitive, and emotional symptoms such as headaches, dizziness, confusion, oculomotor deficits, memory disturbances, and impaired executive functioning [4, 5].

Diagnosis and assessment typically involve a combination of clinical evaluation and imaging techniques, with brain computed tomography (CT) scans being the most common tool for detecting intracranial abnormalities such as hemorrhages and fractures [5].

This study aims to identify specific predictors associated with the presence of brain CT abnormalities in patients presenting with mTBI. Understanding these predictors is critical for optimizing the risk vs. benefit of CT scans, enhancing patient outcomes, and effectively allocating healthcare resources.

## Methods

This study is a retrospective analysis of an observational cohort of adult patients who presented to the emergency department (ED) of a level I trauma facility with a mild traumatic brain injury (mTBI), defined by a Glasgow Coma Scale (GCS) score of 13–15. Eligible patients were those whose mTBI occurred within 24 h prior to their ED presentation, from any mechanism, including fall, motor vehicle collision, assault, or recreational or sports-related injury. Written informed consent was obtained from participants for the inclusion of their data in the study, which was approved by our institutional review board.

The Glasgow Coma Scale (GCS) is a standardized tool used to assess the level of consciousness in patients with head injuries [6]. It consists of three components: eye opening (scored 1–4), verbal response (scored 1–5), and motor response (scored 1–6). The total score ranges from 3 to 15, with higher scores indicating better neurological function. A score of 13–15 is classified as mild TBI, 9–12 as moderate TBI, and 3–8 as severe TBI. The GCS is widely used in clinical practice to quickly evaluate the severity of brain injuries and guide management decisions [7].

Data was collected on various demographic and clinical variables, including patient age, sex, mechanism of injury, initial GCS score, and the presence of clinical symptoms such as headache, vomiting, loss of consciousness, post-traumatic amnesia, and focal neurological deficits. Additional data included the results of brain CT scans, which were assessed for abnormalities such as intracranial hemorrhage, contusions, fractures, and other signs of significant injury.

Data management and statistical analyses were conducted using JMP 16.0. Regression analyses were performed to identify predictors of CT scan abnormalities, with results expressed as odds ratios (OR), and associated p-values with 95% confidence intervals (CI).

## Results

A total of 2786 patients were in the cohort, of which 2548 (91%) had a brain CT and 698 (27% of imaged cohort) demonstrated abnormal findings. As this was an observational study, whether a patient had a head CT performed was at the discretion of the treating Emergency Physician. Table 1 delineates the characteristics between those who had a head CT done and those who did not. The 9% that did not have a head CT done were significantly more often male, younger, did not have peri-injury symptomatology, were more likely to have a GCS of 15, and were less likely to have sustained their TBI from a fall or motor vehicle collision mechanism.

**Table 1:** Cohort characteristics

	Head CT performed (*n* = 2548)	NO head CT performed (*n* = 238)	*P*-value (z test for proportions)
Sex	0.59	0.42	*P* < 0.00001
Median age	40 years	27 years	
Fall mechanism	0.51	0.37	*P* < 0.00001
MVC mechanism	0.33	0.36	NS
LOC	0.49	0.03	*P* < 0.00001
AOC	0.31	0.12	*P* < 0.00001
Associated seizure	0.02	0.01	NS
Associated vomiting	0.06	0.03	NS
PTA	0.29	0.07	*P* < 0.00001
ED GCS 13	0.03	0	*P* = 0.0067
ED GCS 14	0.11	0.02	*P* < 0.00001
ED GCS 15	0.86	0.98	*P* < 0.00001

The most frequently observed CT abnormalities were bleeding (*n* = 638, 91% of abnormal scans, or 25% of imaged cohort) and fractures (*n* = 190, 27% of abnormal scans or 7.4% of imaged cohort). Extrapolating these results to the 9% of the cohort that did not get a brain CT (*n* = 238), 60 bleeds and 18 fractures were potentially missed, although that cohort was significantly different in many ways as highlighted above.

**Fig. 1:**
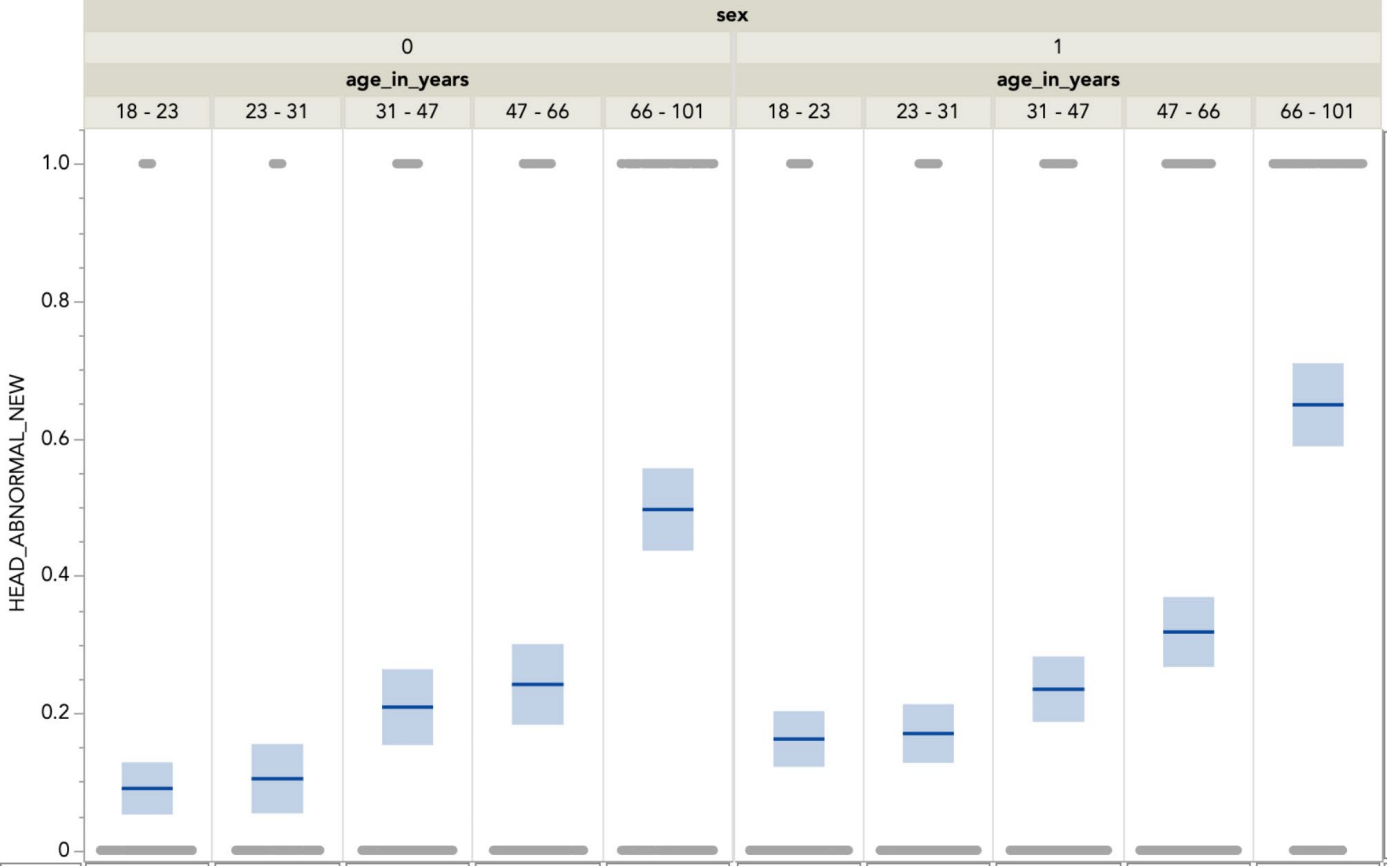
Distribution of Brain CT Abnormalities by Age Groups and Sex (0 = female, 1 = male)

Figure 1 illustrates the distribution of brain CT abnormalities across different age groups and sexes. The incidence of abnormalities increases with age, with the highest occurrence observed in the 66–101 age group, which had the highest occurrence of CT-detected abnormalities. This trend underscores the heightened vulnerability of older adults to sustaining more severe intracranial injuries following mild TBI. Furthermore, males exhibited a consistently higher incidence of abnormalities across all age groups compared to females, reflecting the underlying sex-based difference in injury mechanisms or possibly physiological responses to trauma. The figure also highlights that younger age groups, particularly those aged 18–23 and 23–31, show the lowest incidence of abnormalities, suggesting that they may have a greater capacity for resilience or recovery following mild TBI, or that their TBI mechanism was of a more benign nature. The distribution of peri-injury symptomatology is summarized in Table 2.

**Table 2:** Distribution of peri-injury symptomatology

	Did not have it	Had it	Unknown
LOC	1137, 41%	1301, 47%	348, 12%
AOC	1749, 63%	818, 29%	219, 8%
Associated seizure	2733, 98%	49, 2%	0, 0%
Associated vomiting	2617, 94%	167, 6%	0, 0%
PTA	1816, 65%	762, 27%	208, 7%

**Fig. 2:**
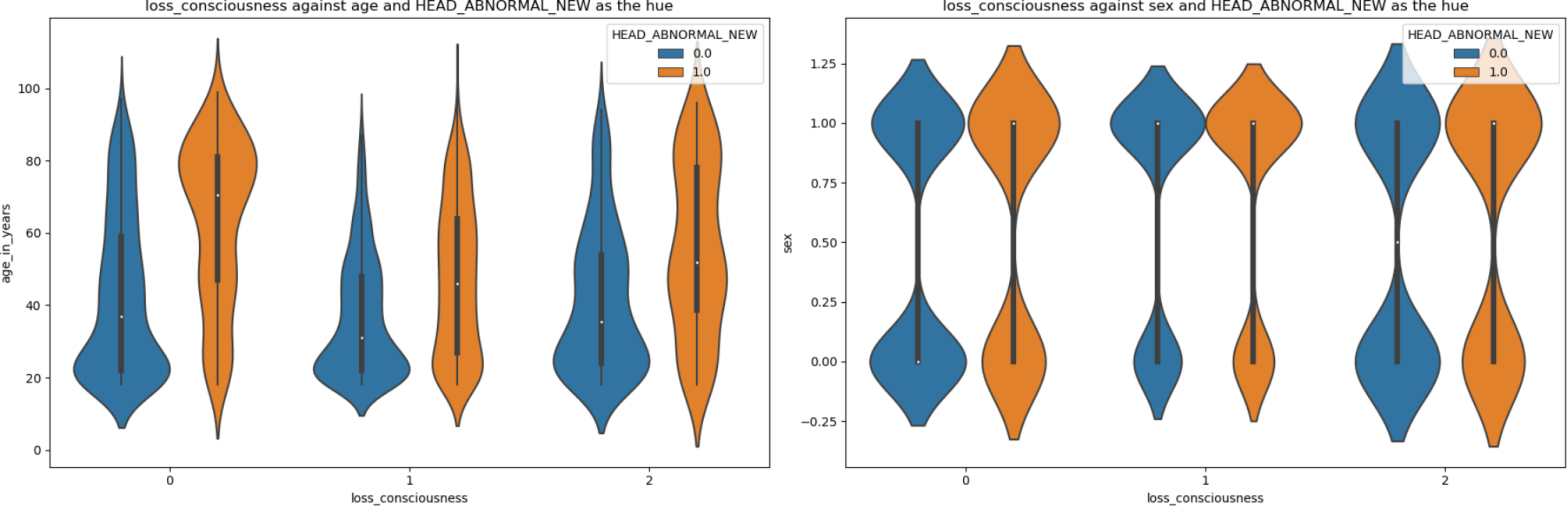
Violin plot depicting the relationship between LOC, age, sex, against the outcome of abnormal head CT. Older patients tended to have an anormal CT scan regardless of the presence of LOC, with the highest population age density centered around 60 to 80 years. The largest difference in median age between normal and abnormal ct scans was for the population where there was no loss of consciousness

**Fig. 3:**
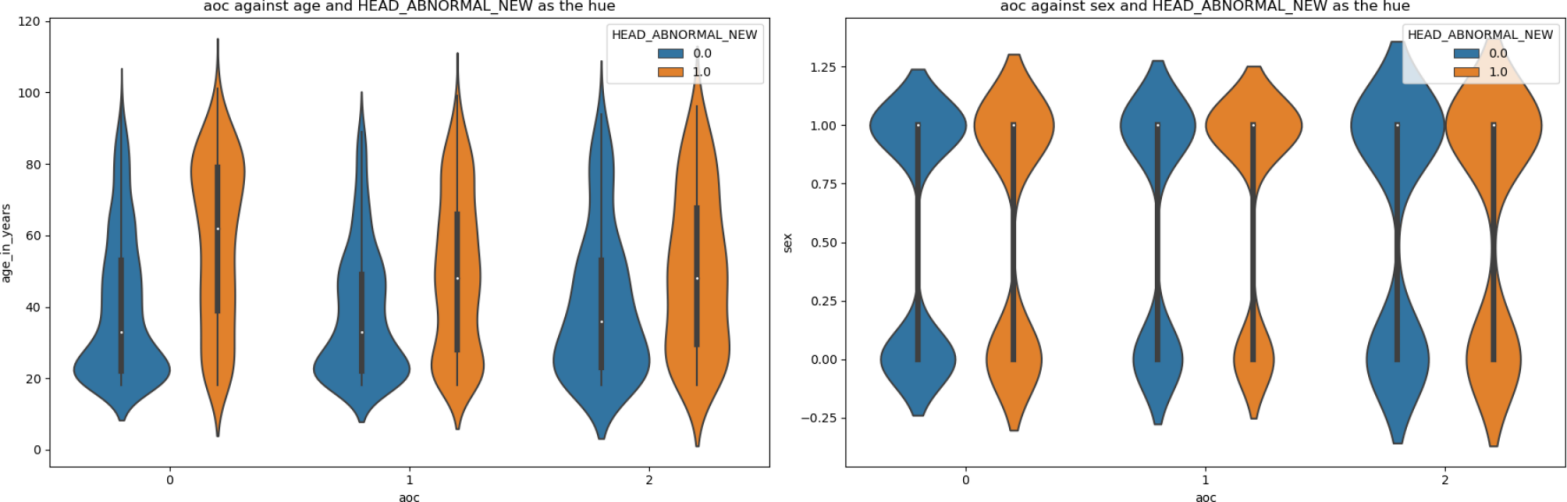
Violin plot depicting the relationship between AOC, age, sex, against the outcome of abnormal head CT. For both sexes, the distribution is somewhat similar, though more data points are represented in AOC unknown than in no AOC or with patients who have AOC

**Fig. 4:**
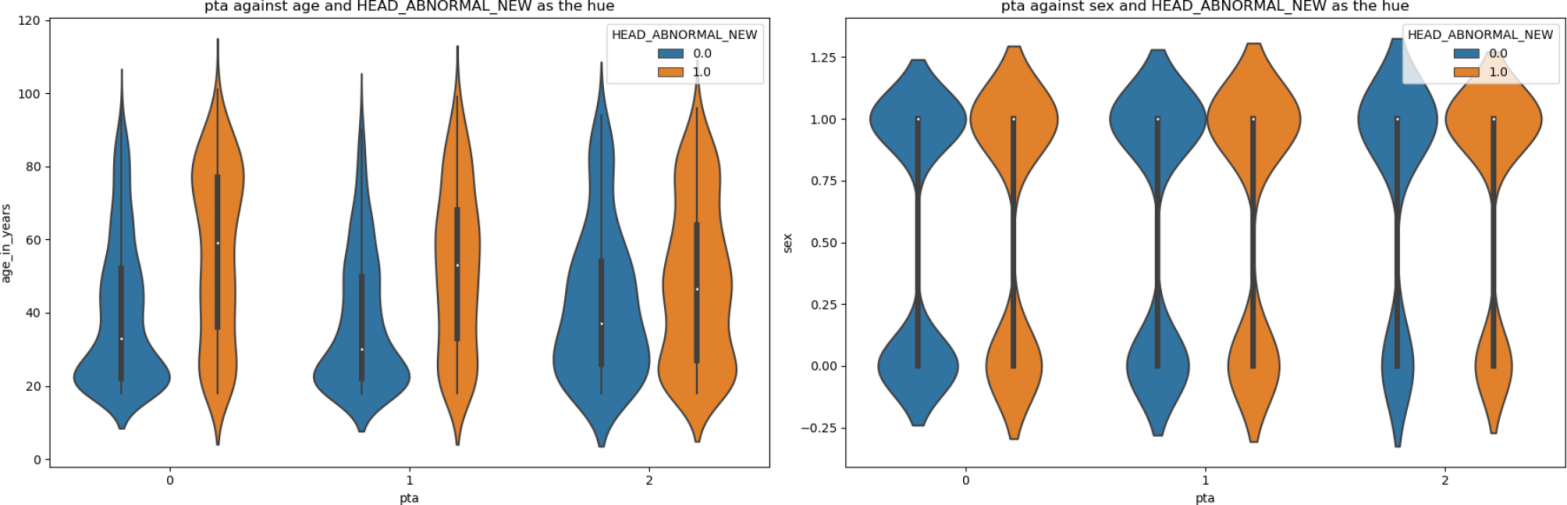
Violin plot depicting the relationship between PTA, age, sex, against the outcome of abnormal head CT. The shapes of the violin plots indicate that while the age distribution of patients with, without, or unknown PTA are comparable. The violin plot shapes suggest that for both sexes, the distribution is somewhat similar, though more data points are represented by males (1) than females (0)

**Fig. 5:**
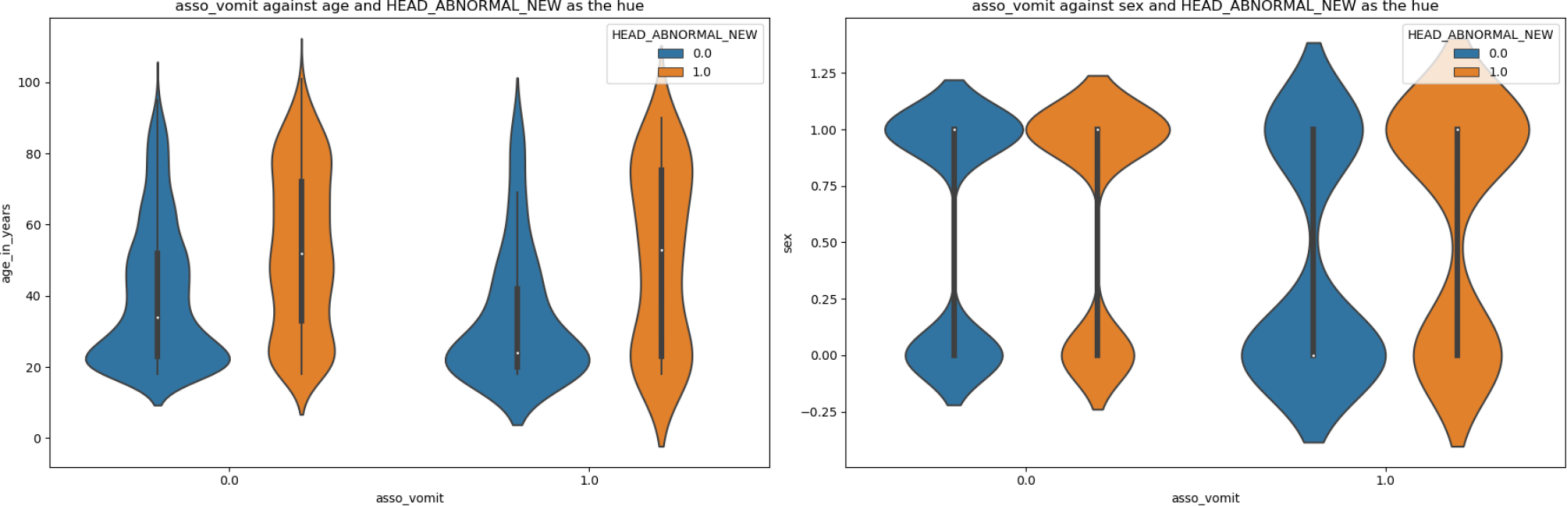
Figure 5: Violin plot depicting the relationship between associated vomiting, age, sex, against the outcome of abnormal head CT. The median age of patients with or without associative vomiting with abnormal CT and is around the age of 50. There was a higher concentration of vomiting and abnormal head CT in the younger and older age groups compared to the middle-aged group. For patients with associated vomiting, more men had an abnormal CT scan than, suggesting that vomiting is a better indicator of an abnormal head CT scan in men rather than women

Figures 2-5 are violin plots that help to visualize the relationships between the peri-injury symptomatology and the outcome of abnormal head CT, depicted by age and sex

A multivariate logistic regression model including age, GCS score, and injury symptomatology, revealed the following significant predictors associated with the presence of brain CT abnormalities (Table 3): older age [*P* < 0.0001], male sex [*P* < 0.0001], loss of consciousness [*P* = 0.0041], associated vomiting [*P* = 0.0011], alteration of consciousness (AOC) [*P* = 0102]. This was a robust model with an R² of 14.2%.

**Table 3:** Multivariate regression model depicting variables significantly associated with having an abnormal brain CT

Term	Estimate	Std Error	Chi-Square	*P*-value	Lower 95%	Upper 95%	Odds ratio	Lower 95%	Upper 95%	Reci-procal
Age	0.0385	0.0023	272.99	< 0.0001	0.0339	0.0430	1.0392	1.0345	1.0440	0.9622
Sex	0.4749	0.1033	21.14	< 0.0001	0.2725	0.6774	1.6079	1.3132	1.9688	0.6219
Associated vomiting	0.6406	0.1966	10.62	0.0011	0.2553	1.0259	1.8975	1.2908	2.7895	0.5270
Associated seizure	0.5202	0.3282	2.51	0.1130	-0.1232	1.1635	1.6823	0.8841	3.2010	0.5944
Loss of consciousness	0.1575	0.0771	4.17	0.0411	0.0064	0.3086	1.1706	1.0064	1.3616	0.8543
Alteration of consciousness	0.2143	0.0834	6.60	0.0102	0.0509	0.3778	1.2390	1.0522	1.4591	0.8071
Post Traumatic Amnesia	0.0847	0.0852	0.99	0.3203	-0.0824	0.2518	1.0884	0.9209	1.2863	0.9188
GCS score in the ED	-0.6211	0.1047	35.17	< 0.0001	-0.8264	-0.4159	0.5373	0.4376	0.6598	1.8611

## Discussion

In this study, we identified several significant predictors of brain CT scan abnormalities in patients with mild traumatic brain injury (mTBI). The analysis revealed that loss of consciousness (LOC), alteration of consciousness (AOC), post-traumatic amnesia (PTA), associated vomiting, and associated seizures were all significantly associated with abnormal CT findings. These results highlight the importance of these clinical factors in the initial assessment and decision-making process for mTBI patients.

These findings are supported by previous studies, which emphasized the significance of neuroimaging in all TBI patients, including those with a GCS of 15. A study of over 2000 patients found that even the mildest cases of TBI could present with significant CT abnormalities, with variables such as LOC, AOC, and PTA being significantly associated with adverse outcomes, including ICU admission and mortality [3]. Similarly, a 2017 study that included 453 patients with GCS 15 found that presence of vomiting or any symptom combination including LOC, bleeding, seizure or vomiting was significantly associated with having an abnormal brain CT (*P* < 0.0001) [8].

Further, these findings highlight the challenges in developing standardized assessment protocols for mTBI in emergency settings, particularly given the heterogeneity of mTBI presentations and the lack of consensus on management guidelines, especially in mild cases [9, 10]. This underscores the need for integrating identified clinical predictors into decision-making processes, which can help standardize and improve the assessment, leading to better patient outcomes.

The most common clinical decision tool used to determine whether a head CT should be done after a mTBI is the Canadian head CT rule [11]. This rule states that if all criteria are negative, then the sensitivity of the rule for any intracranial traumatic finding is 83–100%. The included high-risk criteria are age > 65, suspected skull fracture, signs of basilar skull fracture, or 2 or more episodes of vomiting. Medium risk criteria include 30 or more minutes of amnesia to the event (PTA), and a dangerous mechanism for the TBI, defined as pedestrian struck by motor vehicle, occupant ejected from motor vehicle, or fall from > 3 feet or > 5 stairs. In our cohort, after removing every patient who had even 1 of these criteria, there were still 1309 patients left, or 47%. All of these would be deemed to not need a head CT because traumatic findings would not be expected. Indeed, a quarter of these patients actually did have intracranial hemorrhage, and 7% had a fracture.

One of the key strengths of this study is the large sample size, which provides robust evidence for the identified predictors. The multivariate logistic regression model used in this analysis demonstrated a strong predictive capability, with an R² of 14.2%, indicating a substantial proportion of the variance in CT abnormalities can be explained by the included predictors. The inclusion of a wide range of clinical variables enhances the comprehensiveness of our analysis, ensuring that the identified predictors are not confounded by other factors.

Despite these strengths, it is crucial to acknowledge the limitations of this study. CT scans primarily offer structural information and may not detect functional abnormalities, which are often critical in the assessment of mTBI. Functional changes in brain activity, which can significantly impact cognitive function, are not directly observable on CT scans. Additionally, the timing of CT scans may affect the detection of abnormalities. Early CT scans might miss evolving injuries that become apparent only later, emphasizing the potential need for follow-up imaging and more frequent monitoring of mTBI patients.

The identification of intracranial hemorrhage on CT in mTBI is important, even if neurosurgical intervention is not performed. A 2021 study in JAMA Neurology [12] reports that patients who sustained a subarachnoid hemorrhage, epidural hematoma or subdural hematoma were significantly more likely to have incomplete recovery from their TBI, even at 1 year. This information is important to frame prognosis and make rehabilitation plans for the patient.

The findings from this study have significant implications for clinical practice. Incorporating the identified predictors into clinical decision rules could improve the accuracy of triaging mTBI patients, ensuring that those at higher risk receive timely and appropriate imaging and care. This targeted approach can optimize resource allocation in emergency departments, reducing unnecessary CT scans for low-risk patients while prioritizing high-risk cases. Future research should focus on developing and validating clinical decision rules that integrate these predictors, potentially incorporating advanced imaging techniques and functional assessments to enhance the diagnostic process for mTBI.

Overall, this study underscores the critical role of specific clinical predictors in identifying brain CT abnormalities in mTBI patients. These findings contribute to the growing body of evidence that informs clinical guidelines and decision-making protocols, ultimately aiming to improve patient outcomes and healthcare efficiency. By integrating clinical predictors with neuroimaging and understanding the broader context of TBI management, clinicians can adopt a more comprehensive approach to diagnosis and treatment, hopefully leading to better outcomes for mTBI patients.

## Conclusion

In this retrospective analysis, loss of consciousness, altered level of consciousness (AOC), post-traumatic amnesia (PTA), associated vomiting, and associated seizures were identified as significant predictors of abnormal brain CT findings in patients with mild traumatic brain injury (mTBI). These results emphasize the importance of considering these clinical factors when assessing the need for brain CT scans in mTBI patients. Early identification of individuals at higher risk for CT abnormalities can aid in optimizing patient management and the efficient allocation of healthcare resources in the emergency department setting. Moreover, these findings could contribute to the development of more refined clinical decision rules, enhancing the accuracy of diagnostic evaluations for mTBI patients.

## Data Availability

No datasets were generated or analysed during the current study.
